# The Coevolution of Plants and Microbes Underpins Sustainable Agriculture

**DOI:** 10.3390/microorganisms9051036

**Published:** 2021-05-12

**Authors:** Dongmei Lyu, Levini A. Msimbira, Mahtab Nazari, Mohammed Antar, Antoine Pagé, Ateeq Shah, Nadia Monjezi, Jonathan Zajonc, Cailun A. S. Tanney, Rachel Backer, Donald L. Smith

**Affiliations:** 1 Department of Plant Science, Macdonald Campus, McGill University, Montreal, QC H9X 3V9, Canada; Dongmei.Lyu@mail.McGill.Ca (D.L.); Levini.Msimbira@mail.McGill.Ca (L.A.M.); mahtab.nazari2@mail.mcgill.ca (M.N.); Mohammed.Antar@mail.McGill.Ca (M.A.); Antoine.Page@cnrc-nrc.gc.ca (A.P.); atiqshah88@yahoo.com (A.S.); Nadia.Monjezi@mail.McGill.Ca (N.M.); Jonathan.Zajonc@mail.McGill.Ca (J.Z.); Cailun.Tanney@mail.McGill.Ca (C.A.S.T.); rachel.gm.backer@gmail.com (R.B.); 2 National Research Council Canada, Aquatic and Crop Resource Development (ACRD), Montréal, QC H4P 2R2, Canada

**Keywords:** plant evolution, phytomicrobiome, symbiosis, pathogenic interaction, holobiont

## Abstract

Terrestrial plants evolution occurred in the presence of microbes, the phytomicrobiome. The rhizosphere microbial community is the most abundant and diverse subset of the phytomicrobiome and can include both beneficial and parasitic/pathogenic microbes. Prokaryotes of the phytomicrobiome have evolved relationships with plants that range from non-dependent interactions to dependent endosymbionts. The most extreme endosymbiotic examples are the chloroplasts and mitochondria, which have become organelles and integral parts of the plant, leading to some similarity in DNA sequence between plant tissues and cyanobacteria, the prokaryotic symbiont of ancestral plants. Microbes were associated with the precursors of land plants, green algae, and helped algae transition from aquatic to terrestrial environments. In the terrestrial setting the phytomicrobiome contributes to plant growth and development by (1) establishing symbiotic relationships between plant growth-promoting microbes, including rhizobacteria and mycorrhizal fungi, (2) conferring biotic stress resistance by producing antibiotic compounds, and (3) secreting microbe-to-plant signal compounds, such as phytohormones or their analogues, that regulate aspects of plant physiology, including stress resistance. As plants have evolved, they recruited microbes to assist in the adaptation to available growing environments. Microbes serve themselves by promoting plant growth, which in turn provides microbes with nutrition (root exudates, a source of reduced carbon) and a desirable habitat (the rhizosphere or within plant tissues). The outcome of this coevolution is the diverse and metabolically rich microbial community that now exists in the rhizosphere of terrestrial plants. The holobiont, the unit made up of the phytomicrobiome and the plant host, results from this wide range of coevolved relationships. We are just beginning to appreciate the many ways in which this complex and subtle coevolution acts in agricultural systems.

## 1. Introduction

The sophisticated and complex association between plants and microorganisms, including bacteria and fungi, have existed since the early stages of life on Earth. Colonization of terrestrial habitats began with plants, followed by animals, and was possible only when specific genes from terrestrial bacteria were transferred to algae, in order to increase tolerance to abiotic and biotic stresses present on land [1]. The relationship of cyanobacteria, a prokaryote, with eukaryotes that eventually developed into algae, was a pivotal step in the progression of this evolution [2]. Cyanobacteria played a pivotal role in formation of algae through endosymbiosis by which a cyanobacterium was incorporated into a heterotrophic eukaryote ancestor where it was retained and specialized into an organelle, thus bringing about photosynthetic eukaryotes [3]. Molecular multi-gene phylogeny has clearly indicated that cyanobacteria became the primary plastid in green and red algae, and glaucophytes; there has also been a series of secondary endosymbiosis with other eukaryote ancestors [4]. The colonization of land plants by fungal and bacterial symbionts was a critical stage to bringing about evolution of terrestrial ecosystems, but how the members of early communities interacted and influenced one another is still relatively unexplored [5]. An expanding body of fossil evidence shows that interactions among early terrestrial communities included bacteria, fungi, algae, lichens, and bryophytes—the ecosystem services provided by these organisms include the weathering of parent rock material, soil formation, stabilization of sediments, and the productivity of ecosystems [6,7].

When plants moved onto the land, the role of the microorganisms became clearer, including improving plant tolerance to biotic and abiotic stresses. Mutualistic interactions have been reported among microbes (e.g., plant growth promoting bacteria (PGPB) and mycorrhizal fungi) which enhance nutrient acquisition, control elements of plant development and physiology through signal compounds and phytohormones/hormone analogues that trigger stress resistance in the host plant, and by producing compounds with antagonistic activity toward plant pathogens [8,9,10]. In turn the associated plant provides habitat and releases exudates into rhizosphere, including reduced carbon as an energy source for the growth of phytomicrobiome members. Through the interactions between microorganisms and associated plants, the two together form the holobiont [11].

In this paper we focus on the role of microorganisms in the evolution of plants. We first discuss the endosymbiosis of microbes which lead to the evolution of critical plant organelles. Then, we review the fundamental roles of microbes in plant development and survival, from mutualistic to parasitic/pathogenic interactions. Finally, we propose that the holobiont concept should be incorporated into thinking around agricultural systems based on the role of microbial communities in agro-ecosystems.

## 2. The Role of Microorganisms in the Evolution of Plants

### 2.1. Endosymbiotic Evolution

During the course of evolution, eukaryotes (organisms with subcellular organelles, e.g., plants, animals, protozoans) were derived from prokaryotes (organisms without subcellular organelles, e.g., cyanobacteria and bacteria) through multiple steps [12,13]. The first evolutionary steps were the development of aerobic prokaryotic microbes, such as the protomitochondrion, and their survival in the newly oxygen-containing atmosphere. For example, as Burki [14] reported, one of the defining events in the evolution of eukaryotes was the origin of mitochondria by the endosymbiotic incorporation of an α-proteobacterium. The evolution of the first amitotic amoeboid aerobic species resulted from this obligatory endosymbiosis process [13,15,16]. While the specifics of this endosymbiosis are still debatable, investigations over the past 20 years in cell biology and molecular evolution have shown that the origin of mitochondria is closely related to the divergence of all known eukaryotes. This indicates that all surviving eukaryotes, or at least their ancestral lineages, are expected to have mitochondria in one form or another [15,16], unless they have been shed in extreme parasitic relationships, such as that of *Giardia* [17]. Another fundamentally important step in the evolution of plants was the endosymbiosis of photosynthetic cyanobacteria that resulted in chloroplasts [18,19]. This occurred more than once (via primary and secondary endosymbiosis) and some intermediates remain extant [20]. While large-scale gene-loss from the cyanobacterial symbionts has taken place throughout the evolution of the chloroplast, key cyanobacterial functional aspects have been retained [21].

For evolutionarily stable endosymbiotic relationships, there must be a selective advantage in which the pair in the relationship is selected for over individually reproducing groups. Cells of different lineages must have aligned their capacities for evolutionary success and develop synergies based on their differing characteristics to achieve a stable endosymbiosis [22,23]. The importance of mitochondria and chloroplasts are paramount for plant growth and development. Chloroplasts convert CO_2_ to carbohydrates, conduct the synthesis of amino acids and fatty acids, and serve as the site for reduction of nitrite (NO_2_^−^) to ammonium, for incorporation into organic compounds [24] and sulphate assimilation [25]. Mitochondria, are critical for plant respiration, using alternative electron acceptors to generate ATP supplied to a wide range of metabolic reactions [26]. It is clear that both of these critically important organelles evolved from microbes and have been fundamental to plant evolution. The origin of mitochondria and plastids is quite certain since their genomes, and the expression and arrangement of harbored genes of these organelles, are clearly derived from the bacterial classes α-proteobacteria and cyanobacteria, respectively [27]. As a result, the DNA sequences of plant tissues shares some similarities with DNA sequences of cyanobacteria [28]. This explains why it is difficult to amplify bacterial marker genes in plant tissue. However, it also serves as a conceptual bridge between the phytomicrobiome and the plant, both of which make up the holobiont [11].

### 2.2. Relationships between Microbes and the Precursor of Terrestrial Plants: Green Algae

**Figure 1:**
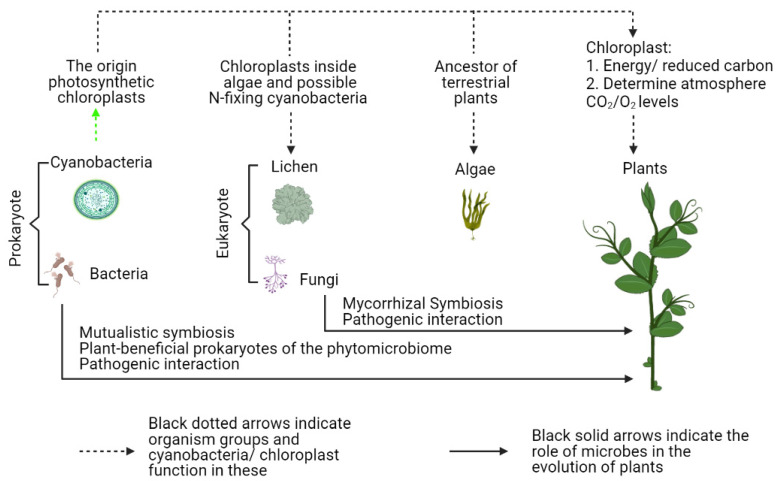
A range of microorganisms contribute to plant function, allowing them to become the dominant terrestrial primary producers, such as crop plants. This includes prokaryotes becoming organelles (mitochondria and chloroplasts), with cyanobacteria focused on because of their pivotal role in giving plants photosynthetic ability, through chloroplasts, and a range of other microbes forming other beneficial (plant growth promotion microbes) and negative (pathogens) associations with plants. The combination of fungi, algae (with cyanobacteria-derived chloroplasts) and, at least sometimes, cyanobacteria, resulted in a parallel “organismal” development.

Green algae are the precursor of terrestrial plants, which have lived in freshwater habitats for hundreds of millions of years. These habitats can include shallow puddles, riverbeds and rocks protruding from freshwater sources, all of which can occasionally became quite dry; extreme environmental events likely shaped the relationships between algae and bacteria [29,30]. While bacteria and algae interact in a variety of ways (mutualism, commensalism and parasitism/pathogenicity) [2], many studies have revealed mutualisms between algae and bacteria. Often, algae provide fixed organic carbon to bacteria, mostly PGPB, and in return, bacteria provided dissolved inorganic carbon, mineral nutrients, or vitamin B_12_ required for algal growth [31,32,33]. In a specific example, a mutualistic relationship between a well-known PGPB, *Rhizobium* sp., and a wastewater alga, *Chlorella vulgaris*, enhanced algal growth approximately 72% [34]. It also appears that mutualistic interactions between algae and fungi resulted in improved nutrient and water uptake by algae. As a result, mycorrhizal genes were taken up by the ancestors of land plants, probably algae, and their functions were effectively protected/retained during all of land plant evolution [35]. Taken together, all of this indicates that the interactions between algae, bacteria, and fungi comprise a sophisticated network of associations among pioneers of the terrestrial habitat, resulting in evolution of terrestrial plants (Figure 1).

### 2.3. The Coevolution of Terrestrial Plants and Microorganisms

#### 2.3.1. Microbially-Drived CO_2_ Fixation

Land colonization by plants introduced them to an environment that possessed abundant levels of resources, such as CO_2_, nitrogen, and phosphorus, to support the full potential of photosynthesis, compared with the resource limited aquatic plants [36,37]. Initially, all cells depended on external sources of organic compounds to satisfy their metabolic needs. Gradually, when resources became limited, competition for survival intensified resulting in selection that favored those with lower requirements for materials from the external environment. Over the course of time, cells with the ability to use simple inorganic molecules to form energy-rich compounds needed for their survival evolved [36], allowing protracted evolution of life rather than the process ending due to exhaustion of key resources. When there is a surplus of resources, evolutionary pressures relax and a greater amount of diversity is tolerated. For instance, the higher CO_2_ concentrations on land than in water contributed to more photosynthesis by plants, but the shift also brought the long-term cost of photorespiration since photosynthesis eventually led to increased O_2_ levels and decreased CO_2_ levels in the atmosphere [38,39]. Ribulose-1,5-bisphosphate carboxylase/oxygenase (Rubisco) plays a pivotal role in carbon fixation during oxygenic photosynthesis, and has become more effective at this during the course of evolution [40,41]. Reduced atmospheric CO_2_ levels and increased O_2_ levels resulted in higher rates of O_2_ reduction by Rubisco (photorespiration), which required greater substrate specificity leading to slower CO_2_ fixation by Rubisco [42,43]. Rubisco initially evolved in free-living cyanobacteria and then continued to fix carbon in the chloroplasts in a wide range of photo-autotrophic eukaryotes [43,44], so that cyanobacteria brought Rubisco to plants. Rubisco and the CO_2_ fixation cycle are absolutely essential for life, as it currently exists on earth. The evolution of Rubisco and the associated photosynthetic capacity provided by endosymbionts have had a profound effect on the earth and its atmosphere. Interestingly, because Rubisco is a large protein and is in high concentrations in plant leaves, it is the most abundant protein in leaves [45] and the most abundant protein globally [46]; it is a critical source of protein for herbivores and, interestingly, has an exceptionally good amino acid balance for herbivores [47]; through the efficiencies of having an amino acid profile matching the protein source microbes (cyanobacteria), by becoming plant Rubisco-chloroplasts, probably influenced/drove the amino acid composition of herbivorous animals. Additionally, in extreme environmental condition or under situations of limited resource supplement, plant survival also depends on the microbes to obtain what they need through building and maintain key symbiotic relationships and managing pathogenic ones [48].

#### 2.3.2. Microbially-Facilitated N and P Acquisition

The evolution of terrestrial plants from the aquatic environment brought a plethora of challenges. However, with time, plants coevolved with other living entities to overcome the new and challenging circumstances. Major constraints faced by the earliest land plants, and to this day are still of major concern, were inadequate water and nutrient supply. Plants, in addition to light and water, need adequate supply of essential nutrients to fuel their metabolic machinery for growth and survival, and a solution to this requirement was to evolve symbiotic relationships with soil microbiota (e.g., rhizobacteria and arbuscular mycorrhizal fungi, [7]). In fact, before the establishment of symbiotic relationships between terrestrial plants and microorganisms, lichens, as a classic example of a symbiosis involving algae, were amongst the early colonizers of many landscapes, adapting to severe conditions in pre-vascular plants. They inhabit rocks or sandy soil and make substrates for mosses and vascular plants available through the production of secondary metabolites, collectively referred to as lichen acids [49,50].

Legume-*Rhizobium* symbioses constitute the most extensively studied and well-understood belowground plant-microbe interaction. More than 70% of legumes develop symbiotic relationships with rhizobia [51]. This association begins with crosstalk between both partners through chemical signals. Isoflavonoids exuded from legumes are perceived by rhizobia through NodD (LysM-RLK) receptors, activating *nod* genes. In response to plant signals, rhizobia secrete a combination of Nod factors (LCOs) and effector proteins, which are perceived by Nod factor-specific (LysM-RLK) receptors in plants [10]. In the same way, chemically distinct LCOs are secreted by rhizobia, to which only specific legume species respond. The secretion of correct signals by both partners is crucial in establishing successful associations. The Nod factors received by plant receptors initiate the symbiosis signaling pathway, leading to deformed root hairs, root hair curling, and the formation of infection threads for bacterial entrance into host root cells, ultimately forming specialized endosymbiotic root nodules [52]. Within the nodules, rhizobia differentiate into nitrogen fixing symbiosomes, but unlike chloroplasts and mitochondria they have not been fully integrated into the plant and its genome, and are not inherited by successive plant generations.

Another well studied plant-microbe symbiosis occurs with mycorrhizal fungi. These associations are probably the most widespread mutualistic symbioses, forming with 70–90% of plant species [53,54]. These relationships are estimated to have appeared 450 to 530 million years ago [55,56]. The fossil record indicates the presence of mycorrhizal associations at the very start of, and throughout, the plant transition to land [35]. In this relationship, mycorrhizal fungi significantly improve plant access to nutrients, such as phosphorus (providing up to 70% of plant P requirements) by limiting root cortical cell growth, and increasing root exploration of soil via the development of a hyphal network that can be hundreds of times longer than root hairs [57,58]. Since mycorrhizal fungi can only transport soluble phosphorus, these fungi can work in tandem with phosphorus-solubilizing rhizosphere bacteria to gain access to the large insoluble phosphorus pool in soil [59,60]. In return, mycorrhizal fungi receive approximately 20% of plant photosynthates, which in total accounts for the consumption of approximately 5 billion Mg carbon year^−1^. Plant-mycorrhizae associations are established via signal exchange between the two partners. Strigolactone is perceived by an unknown receptor in arbuscular mycorrhizal fungi [61] and in response, arbuscular mycorrhizal fungi secrete effector proteins, chitin oligosaccharides and lipochitooligosaccharides (Myc factors) [62], which are perceived by Myc factor receptors in the plants. These compounds activate calcium oscillations within plant cells, eventually leading to suppression of plant innate immunity, which encourages hyphal attachment and penetration of root cortical cells that leads to the formation of specialized endosymbiotic structures known as arbuscules [54]. Unlike plastids and mitochondria, which have become plant organelles, mycorrhiza have maintained their identities in the plant-mycorrhizal symbiosis [36], although mycorrhizal fungi do not usually grow in the absence of the plant. However, several genes required for mycorrhizal–plant relationships were transferred from fungi to the ancestors of land plants, resulting in the retention of mycorrhizal genes and gene functions over the course of land plant evolution [35].

#### 2.3.3. Pathogenic Interactions and the Role of Biocontrol Microbes

The evolution of plants has been accompanied by the evolution of beneficial and pathogenic microbes, all of which play critical roles in modern plant physiology and development. Pathogenic microbes establish complex and diverse intimate relationships with plant hosts to obtain nutrients required for microbial growth and development, thus causing plant infection and disease [63,64]; microbes that cause plant diseases are mainly divided into biotrophs and necrotrophs, which rely on living or dead plant tissue, respectively [64]. Over the course of evolution, plants have developed robust immune systems which confer the ability to resist pathogen infection. These systems include harnessing beneficial bacteria that can contribute to plant defenses against pathogenic microbes. Pathogenic pressure can alter plant root exudates to have more positive effects on the recruitment of biocontrol microorganisms. It can, for example, lead to increased emissions of various organic acids (e.g., succinic acid, malic acid, citric acid, fumaric acid; [65,66,67]), which can, in return, modify the composition of the associated rhizospheric microbiome [68], including via increases in the abundance of well-studied biocontrol bacterial taxa (e.g., *Streptomyces*, *Bacillus amyloliquefaciens*, *Pseudomonas fluorescens*); [69,70,71,72]. Some root exudate components trigger chemotactic responses in specific biocontrol microorganisms. This is, for example, the case of L-malate, L-aspartic acid, L-glutamic acid, L-isoleucine, L-leucine, L-lysine, succinate, and fumarate (*Pseudomonas fluorescens*); [69,70], 1-aminocyclopropane-1-carboxylate (*Pseudomonas putida*); [73], alic acid, citric acid, succinic acid, fumaric acid, etc. (*Bacillus amyloliquefaciens*; [71,72]). Few causal connections between specific plant biochemical releases, biocontrol microorganisms and pathogen suppression have, however, been drawn. In one study highlighting such links, Liu et al. [74] demonstrated that cucumber tryptophan root exudation promotes colonization by a biocontrol *Bacillus amyloliquefaciens* strain over that of the pathogen *Fusarium oxysporum*. Wang et al. [75] showed that tomato root exudations of lactic acid and hexanoic acid favor growth of a biocontrol *Bacillus cereus* strain while reducing the infection rate of *Ralstonia solanacearum*. Ankati et al. [76] also pointed out a similar role for benzoic acid and salicylic acid root exudations with regard to recruitment of a *Sclerotium rolfsii*-suppressing *Pseudomonas* species by *Arachis hypogaea*, while Lakshmanan et al. [77] indicated that malic acid secretion plays a role in the recruitment of *Pseudomonas syringe*-suppressing *Bacillus subtilis* in *Arabidopsis*.

In addition, studies in mono-cropped soils that are continuously infested with the same plant pathogens over several years often show parallel accumulations of biocontrol microbes and plant pathogen resistance [78,79,80]. This phenomenon may be driven by the selection of biocontrol microbes in response to root exudation by the plant that is altered in response to long-term pathogen pressure [81]. The mechanisms underlying the evolutionary struggle between plants, pathogenic microbes, and biocontrol microbes are not yet fully understood. A better understanding of the means and timelines of pathogenic counterstrikes will help us move toward optimal development and application of new microbial biocontrol applications.

## 3. Understanding the Plant Holobiont Will Improve Sustainable Agriculture

Recently, microbial research is gaining popularity due to the critical importance of microbes in agriculture, food science, biotechnology, and human health [82]. Although the importance of microbes in the field of agriculture is catching up with the pace of the research on guts microbes in mammals, the awareness and perceived value are far still behind. Microbes in the rhizosphere have been compared to the mammalian gut microbiomes: both are essential for host survival and health [83]. In fact, microbial members of the rhizomicrobiome are increasingly recognized for their beneficial impacts on plant systems, with the understanding that we have only scratched the surface of both their diversity and roles in development. During the course of evolution, plants have evolved strategies to exchange chemical signals with microbes which confers the ability to (1) protect against pathogen attacks, (2) take advantage of functions provided by beneficial microbes (ranging from nutrient acquisition to immune system activation), and (3) recruit anti-pathogen microbes [48]. As we argued in a previous paper [11], plants are best conceptualized as holobionts, which takes into account the phytomicrobiome and organelles (mitochondria and chloroplasts; entrained microbes) that are essential to plant survival [84]. Microbial evolution has developed and established links between microbes and larger (eukaryotic) organisms, from individual plants to entire agro-ecosystems [85]. The agricultural systems of the future will undoubtedly be designed to take advantage of these capacities. Lessons can, in this regard, be drawn from pathogen resistance breeding programs. Many crop varieties have been developed to resist specific pathogens by introduction of traits from ancient or wild relatives into modern commercial varieties [86,87]. Interestingly, currently selected plant disease resistance traits are largely associated with alterations of crop-plant metabolism, while some may actually stem from improvements of interactions that enhance microbial biocontrol [88,89]. Although breeding efforts have been very successful [90,91], cases of resistance breakdown tend to emerge within a few years of release of a new variety. The latter occurrences are attributed to a range of phenomena, including pathogen evolution to renewed virulence [92,93,94]. Strategies that rely on the sequential deployment of crop varieties with diverse mechanisms of pathogen resistance do, however, show promising results [95]. What may lead to more robust resistance, however, would be the investigation of biocontrol microbes isolated from unrelated plant species applied to established agricultural groups. The idea of introducing unfamiliar microbes, and thus their unique applications toward resistance pathways, may help abate the disease prevalence plaguing monocultures. Endeavors that seek to develop novel microbial biocontrol applications should therefore also aim to design complementary tools (e.g., using plant breeding techniques and/or beneficial microbe technologies that elicit plant immune responses) for pathogen resistance.

Plant-associated microbial communities are dynamic and respond to the ecosystem; rhizosphere microbial abundance is enormous, generally ranging from 20 to 100 × 10^6^ bacteria and more than 10^5^–10^6^ fungi in every 1 g of productive soil [96,97,98]. In addition to the rhizomicrobiome, the phytomicrobiome also includes stem, leaf, and flower endophytes and above ground non-endophytic microbes. The microbial diversity associated with host plants is distinct among species of the same family and even between cultivars of the same species [99,100,101] and the phytomicrobiome community composition is also affected by soil type, available nutrients in the rhizosphere, and other root zone properties (e.g., pH, soil texture, moisture content) [99,102]. Only a limited number of phytomicrobiome interactions have been studied in depth, to characterize symbiotic relationships in only a few land plant lineages; the results of these studies indicate that there are a wide range of these relationships that have not yet been characterized. In addition, plant evolution was also driven by interactions with parasitic and pathogenic microbes [48]. Even when the rigidly symbiotic legume-rhizobia and plant-mycorrhizal fungi relationships are excluded, the large and coordinated phytomicrobiome population is indispensable to plant growth. The highly complex relationships that occur in the rhizosphere are indicative of the intricacy of the holobiont itself, which results from the long coevolution of plants and the phytomicrobiome. The dependencies between plants and microbes have almost certainly increased through the course of evolution, potentially reducing the number of individuals that can grow independently.

As a result, plant-microbe interaction mechanisms have only been marginally explored, in part because the vast majority of microbes, on the order of 95–99%, are non-culturable under the artificial conditions [103]. Specifically, the vast majority of bacteria found in the soil are uncharacterized. One can go into a backyard, sample the plants, and have a very good chance of finding unknown microbial species with equally unknown effects on the phytomicrobiome they came from. The lack of exploitation of bacteria from the holobiont stems from the generally poor understanding of plant-microbe interactions within the holobiont as a whole, due to there being so many unculturable bacteria unavailable for study. The high levels of complexity involved in holobiont relationships, between the plant host and bacteria, may have led to their dependency on each other continuing to increase as they co-evolved. This may have been due to an “arms race” of sorts between pathogenic microbes and other members of a holobiont. Under continued pathogen pressure more nuanced plant-microbe interactions may have arisen, to combat them, leading to beneficial microbes being incorporated into the holobiont’s evolutionary progression, to the point where their survival is best when in direct contact with the holobiont community and vice versa. Close relationships with, and strong dependencies on, the associated plant have driven the substantial number of unculturable bacteria predicted to be within the holobiont system. The use of metagenomic and transcriptomic studies will shed light on (1) the members of the phytomicrobiome community throughout the lifecycle of the plant and (2) what role this community plays in regulating plant growth, development, and immunity to plant pathogens. What we can say for certain, is that the long-standing relationships between plants and microbes underpin key functions, such as plant defenses against pathogenic microbes. As a result, plant holobionts have an adaptive evolutionary advantage in the face of pathogen pressures designed to manipulate the host plant for the pathogen’s benefit. An improved understanding of the phytomicrobiome will lead to progress in crop production under stressed or unstressed growing environments and the plant holobiont paradigm will allow for the development of innovative and ecologically intensive agriculture [104].

Notwithstanding the success in boosting agricultural productivity through using ever more fertilizer and pesticides, there is a hidden downside of the Green Revolution as it reduces soil microbial diversity as well as negatively impacting plant-microbe interactions through direct and indirect effects [105]. A detailed retrospective of the nutrient management practice, its limits in terms of soil microbial abundance, diversity and activity under long-term fertility management shows that continuing fertilization will unquestionably speed up soil acidification and decrease base saturation, cation exchange capacity, soil aggregation and water storage capacity, which is closely linked with diminished soil microbial diversity [106]. Intensively managed agricultural systems have an intrinsic reliance on agrochemicals, to overcome pest damage, particularly herbicides, which adversely affected the soil biodiversity. It is still too early to fully understand the full impact of pesticides on beneficial soil microorganisms. However, there is no doubt that these chemical pesticides upset the activities of soil microbes by altering and/or decreasing the functional structure and functional diversity of microorganisms [107]. The combined effects of these agronomic management practices on agroecosystem-associated microbial communities have been less studied, and there is a pressing need for more research in this area.

## 4. Conclusions

Plant biologists have begun to understand that the origin, development, and ultimate success of plants is closely linked to plant interactions with the phytomicrobiome. Early evolution studies have indicated that the first terrestrial organisms evolved from the endosymbiosis of cyanobacteria allowing for the derivation of algae, non-vascular plants, and finally vascular plants. Recent explorations of plant-microbe interactions have demonstrated that plants and microbes are inseparable, having coevolved since the advent of the first plants. Knowledge of how early plants survived the challenges associated with colonizing the terrestrial environment and subsequently evolved goes back to the earlier theory of land sterility during the Precambrian period [108], which underestimated both the past and future role of microbes. Microbes have played a pivotal role in the evolution of plants and there is no doubt that microbes still hold the key to a better future for plant survival in the face climate change and a rapidly growing global agricultural sector; interest in this research area continues to grow. A greater understanding of the remarkable functions performed by the phytomicrobiome and their assistance to plant survival is being revealed and acknowledged. Considering plants and their associated phytomicrobiome as one unit, a holobiont, is a necessary step to sustainability of crop production.

Considering plants as holobionts in breeding systems could yield sustainable results when compared to single or few genes improvements whose character may face environmental/evolutionary challenges, with rapidly fading efficacy. As such today’s domestication and improvement of plants, including consideration of advantages associated with the a holobiont approach is certain to reap the benefits of plant-microbe interactions [109], leading to more ecologically sustainable agriculture. Synthesizing the developments made in understanding coevolution of plants and microbes is essential in the continuing efforts to establish and advance potential areas of research that could increase sustainability in agriculture, and allow sustainable responses as evolving challenges to agricultural sustainability arise. Evidence suggests that from the earliest stages of evolution there has been substantial diversity in the microbial communities associated with plants, at any point in time. The current understanding of plants as part of holobionts is yielding more complex scientific questions which require more holistic approaches to arrive at the best hypothesis. This review is a continued effort to emphasize that microbes are always working with (beneficial) or against (pathogenic) their host, which has the potential to lead to a more resilient holobiont.

## Data Availability

Not applicable.
